# Genome sequence of *Lactiplantibacillus plantarum* 6Y-15 isolated from Chinese pickled chill peppers

**DOI:** 10.1128/mra.00817-25

**Published:** 2025-11-12

**Authors:** Yanyan Gao, Lirong Luo, Yongjun Wu, Shuoqiu Tong, Sheng Li

**Affiliations:** 1 Key laboratory of Plant Resource Conservation and Germplasm Innovation in Mountainous Region (Ministry of Education), College of Life Sciences/Institute of Agri-bioengineering, Guizhou University 71206, Guiyang, Guizhou, China; 2 College of Medical Technology, Chongqing Medical and Pharmaceutical College 372299, Chongqing, China; University of Wisconsin-Madison, Madison, Wisconsin, USA

**Keywords:** *Lactiplantibacillus plantarum*, whole genome sequence, pickled chili peppers

## Abstract

We report the whole genome sequence of *Lactiplantibacillus plantarum* 6Y-15 isolated from pickled chili pepper brine preserved for 6 years in Sichuan Province, southwest China. The genome is 3,385,335 bp in size, with 44.46% GC content, and it consists of one circular chromosome and seven circular plasmids.

## ANNOUNCEMENT

Chinese pickled chili peppers are a traditional lactobacillus-fermented food popular in southwestern China. The fermentation environment primarily consists of chili peppers and salt, typically maintaining a salt concentration exceeding 8% and containing antibacterial capsaicin (1). After pickling and maturation, the total acidity stabilizes around pH 3.5 (2, 3). The lactic acid bacteria strain 6Y-15 was isolated from a 6-year-old pickled chili brine sourced from a farmhouse in Jintang County, Sichuan, China (104.46° E, 30.84° N). Initial screening involved diluting the brine and plating it on 1% CaCO3 supplemented de Man-Rogosa-Sharpe (MRS) agar plates containing 1 g/L capsaicin and incubating it at 30°C for 48 h. Colonies forming distinct calcium dissolution zones were then cultured in liquid MRS medium with 1 g/L capsaicin and 8 g/L NaCl at 30°C. After 15 days, strains exhibiting significant growth (based on OD measurements) were selected for determining lactic acid production by high performance liquid chromatography (HPLC). Among these, strain 6Y-15, which showed the highest lactic acid production (1.1×10⁵ mg/L), was chosen for whole-genome sequencing.

Genomic DNA was extracted from strain 6Y-15 using a magnetic bead-based kit (Cat# T07-100, Majorbio, shanghai, China) and whole-genome sequencing was performed by Majorbio Bio-Pharm Technology Co., Ltd (Shanghai, China). Prior to whole-genome sequencing, the strain was identified as *Lactiplantibacillus plantarum* through morphological and 16S rRNA gene analysis (4). The whole-genome sequencing was utilized on both Illumina and PacBio RS II sequencing platforms. For Illumina sequencing, libraries were prepared with the NEXTflex Rapid DNA-Seq Kit (400 bp inserts) and were used for paired-end sequencing (2×150  bp) on NovaSeq 6000. PacBio libraries were constructed using the SMRTbell Express Template Prep Kit 2.0 (~10 kb fragments) and sequenced on Sequel IIe. The Illumina sequencing platform generated 8,812,860 raw reads to reach a depth of 374.56-fold coverage and 8,710,040 clean reads were filtered by Fastp (v0.23.0) (5). The PacBio sequencing platform generated 1,757,272 raw reads (N*_50_*, 21,391  bp). The PacBio raw reads were assembled into a scaffold using Unicycler (v0.4.8) and corrected by mapping Illumina short sequences onto the assembled genome by Pilon (v1.22) (6). Glimmer v3.02 (7), GeneMarkS (8), tRNA-scan-SE v2.0 (9), and barrnap v0.9 were used to predict the protein-coding genes, plasmid genes, tRNA, and rRNA, respectively. Genome annotations were conducted using the NCBI Prokaryotic Genome Annotation Pipeline (PGAP) v6.7 (10). Default parameters were used for all software.

The complete genome of *Lactobacillus plantarum* 6Y-15 has finally been assembled. It consists of 3,385,335 base pairs with a GC content of 44.46%. The genome includes a circular chromosome and seven circular plasmids. The circular chromosome was 3,198,414 base pairs and contains 3,116 protein-coding genes, 16 ribosomal RNAs (rRNAs), 77 transfer RNAs (tRNAs), 4 non-coding RNAs (ncRNAs), and 62 pseudogenes. Multiple gene copies involved in lactic acid synthesis and stress tolerance were identified (Table 1). This genomic data enriches the genetic repository of fermented food microorganisms and provides insights into LAB adaptation under specific environmental stresses.

**TABLE 1:** Information of genes related to 6Y-15 tolerance characteristic

Function classification	Protein-coding gene name	Locus_tag / Protein_id
Proton transmembrane transporter	Na^+^/H + antiporter	ACM640_14570/XPT59096.1 ACM640_00810/XPT60711.1 ACM640_11615/XPT58546.1
	Potassium channel protein	ACM640_07335/XPT60627.1 ACM640_09570/XPT58189.1
	Potassium transporter Kup	Plasmid_C PX056287/XYL09735.1
Efflux transporter	MATE efflux transporter	ACM640_00365/XPT59346.1
	Fluoride efflux transporter	ACM640_00900/XPT59447.1
	AI-2E family transporter	ACM640_05265/XPT60255.1
	DHA2 efflux transporter permease	ACM640_14690/XPT59118.1
Oxidative defense	Catalase	ACM640_15345/XPT60802.1
	Glutathione peroxidase	ACM640_00930/XPT59453.1
	Glutamate cysteine ligase	ACM640_1032/XPT58310.1
Stress repaired	Clp protease families	ACM640_15380/XPT59242.1 ACM640_03550/XPT59944.1 ACM640_08105/XPT57913.1 ACM640_09335/XPT58144.1
	GroEL/ES chaperonin	ACM640_03310/XPT59898.1 ACM640_03315/XPT59899.1
	DnaK/J chaperonin	ACM640_08610/XPT58012.1 ACM640_08615/XPT58013.1
Lactate related genes	L-lactate dehydrogenase	ACM640_01490/XPT59563.1 ACM640_02110/XPT59681.1 ACM640_04880/XPT60181.1 ACM640_05260/XPT60254.1 ACM640_09475/XPT58170.1 ACM640_10425/XPT58328.1

## Data Availability

The whole-genome sequence of *Lactiplantibacillus plantarum* 6Y-15 has been deposited in databases with GenBank accession numbers CP183776, PX056285, PX056286, PX056287, PX056288, PX056289, PX056290 and PX056291, SRA accession numbers SRR34745576, BioProject accession number PRJNA1222760 and BioSample accession number SAMN46787187.

## References

[1] Ye Z, Shang Z, Li M, Qu Y, Long H, Yi J. 2020. Evaluation of the physiochemical and aromatic qualities of pickled Chinese pepper (Paojiao) and their influence on consumer acceptability by using targeted and untargeted multivariate approaches. Food Res Int 137:109535. doi:10.1016/j.foodres.2020.109535 33233164

[2] Tang J, Wu X, Lv D, Huang S, Zhang Y, Kong F. 2024. Effect of salt concentration on the quality and microbial community during pickled peppers fermentation. Food Chem X 23:101594. doi:10.1016/j.fochx.2024.101594 39040148 PMC11261264

[3] Huang Q, Li C, Wu Y, Tong S, Zhang L, Jin J, Zhu Q, Yan Y. 2025. Impact of pepper varieties on microbial succession and correlation with physicochemical properties and volatile compounds during pickled pepper fermentation. Food Chem X 28:102551. doi:10.1016/j.fochx.2025.102551 40491706 PMC12148400

[4] Lirong L, Li Hongyang WY, Sheng L. 2023. Screening of lactic acid bacteria and analysis of probiotic properties in pickled pepper brine. Food and Fermentation Industries 49:159–165. doi:10.13995/j.cnki.11-1802/ts.034228

[5] Chen S, Zhou Y, Chen Y, Gu J. 2018. Fastp: an ultra-fast all-in-one FASTQ preprocessor. Bioinformatics 34:i884–i890. doi:10.1093/bioinformatics/bty560 30423086 PMC6129281

[6] Wick RR, Judd LM, Gorrie CL, Holt KE. 2017. Unicycler: resolving bacterial genome assemblies from short and long sequencing reads. PLoS Comput Biol 13:e1005595. doi:10.1371/journal.pcbi.1005595 28594827 PMC5481147

[7] Delcher AL, Bratke KA, Powers EC, Salzberg SL. 2007. Identifying bacterial genes and endosymbiont DNA with Glimmer. Bioinformatics 23:673–679. doi:10.1093/bioinformatics/btm009 17237039 PMC2387122

[8] Besemer J, Borodovsky M. 2005. GeneMark: web software for gene finding in prokaryotes, eukaryotes and viruses. Nucleic Acids Res 33:W451–W454. doi:10.1093/nar/gki487 15980510 PMC1160247

[9] Chan PP, Lowe TM. 2019. tRNAscan-SE: searching for tRNA genes in genomic sequences. Methods Mol Biol 1962:1–14. doi:10.1007/978-1-4939-9173-0_1 31020551 PMC6768409

[10] Tatusova T, DiCuccio M, Badretdin A, Chetvernin V, Nawrocki EP, Zaslavsky L, Lomsadze A, Pruitt KD, Borodovsky M, Ostell J. 2016. NCBI prokaryotic genome annotation pipeline. Nucleic Acids Res 44:6614–6624. doi:10.1093/nar/gkw569 27342282 PMC5001611

